# Self-Assembly and Electrical Conductivity of a New [1]benzothieno[3,2-b][1]-benzothiophene (BTBT)-Peptide Hydrogel

**DOI:** 10.3390/molecules28072917

**Published:** 2023-03-24

**Authors:** Anna Fortunato, Rafael Cintra Hensel, Stefano Casalini, Miriam Mba

**Affiliations:** Dipartimento di Scienze Chimiche, Università degli Studi di Padova, Via Marzolo 1, 35131 Padova, Italy

**Keywords:** [1]benzothieno[3,2-b][1]-benzothiophene, peptide, supramolecular hydrogel, peptide-chromophore conjugate, π-gel, electrically conductive hydrogel

## Abstract

The conjugation of small-molecule semiconductors with self-assembling peptides is a powerful tool for the fabrication of supramolecular soft materials for organic electronics and bioelectronics. Herein, we introduced the benchmark organic semiconductor [1]benzothieno[3,2-b][1]-benzothiophene (BTBT) within the structure of a self-assembling amphipathic peptide. The molecular structure of the conjugate was rationally designed to favour π-π stacking between BTBT cores and π-delocalization within the self-assembled architectures. Hydrogels with fibrillar structure were obtained upon self-assembly. Spectroscopic studies confirmed that both hydrogen bonding between peptide segments and π-π stacking between BTBT chromophores are responsible for the formation of the 3D fibrillar network observed by transmission electron microscopy. The hydrogel was successfully deposited on gold interdigitated electrodes and a conductivity up to 1.6 (±0.1) × 10^−5^ S cm^−1^ was measured.

## 1. Introduction

Molecular self-assembly of small π-conjugated molecules (SCM) with optoelectronic properties is a powerful tool for the fabrication of feasible ordered nanoarchitectures for organic electronics [1,2]. In this context, the self-assembly of oligothiophenes [3] or p-phenylenevinylenes [4] has been explored extensively in the literature.

In a common approach, the functionalization of the SCM with a specific self-assembling unit introduces additional non-covalent interactions and allows control over the supramolecular organisation. This approach leads to a robust and well-ordered supramolecular architecture in which the SCMs have specific positions, which in turn may affect the efficiency of electronic coupling. Due to its biocompatibility and ability to undergo self-assembly, the conjugation of biomolecules to SCM has been suggested as a powerful approach [5]. Among the various biomolecules capable of self-assembly into ordered supramolecular architectures [6,7], peptides [8], in particular short peptides [9], stand out for several reasons. Peptides are known to form well-ordered secondary structures through directional hydrogen bonds, and their self-assembling properties can be tuned by a proper selection of the amino acid sequence. For example, sequences that feature an alternation of hydrophilic and hydrophobic amino acids are known to favour the formation of 1D β-sheet structures [10,11]. Furthermore, peptides can be easily synthesised with a plethora of available natural and non-natural amino acids. In addition, they also offer biocompatibility [12,13] and the possibility of aqueous processing of the material [14]. In this context, supramolecular peptide hydrogels have been of particular interest in recent years [15,16,17]. Peptides self-assemble in water to give a 3D fibrillar network that entraps the solvent, yielding a gel. Thus, hydrogelation is a process that easily allows the fabrication of 1D supramolecular structures and soft materials for organic electronics [18]. Hydrogelation of peptide conjugates has been used successfully to obtain soft materials incorporating different SCM such as pyrene [19,20], oligothiophenes [21], naphthalene diimide [22], oligo-para(phenylenevinylene) [23], or diketopyrrolopyrrole [24].

Among SCMs, [1]benzothieno[3,2-b][1]-benzothiophene (BTBT) is a promising small-molecule p-type semiconductor [25,26]. Soluble dialkyl, monoalkyl and aryl derivatives allow for solution processes and combine solubility and high hole mobility featuring good thermal stability [27,28,29,30]. For example, Yuan et al. reported an impressive thin film transistor hole mobility of 43 cm^2^ V^−1^ s^−1^ for the 2,7-dioctyl-substituted BTBT (C8-BTBT) [31]. Despite these important achievements, the development of water-processable and biocompatible BTBT derivatives remains an open challenge. Guler et al. were the first to report two amphiphilic BTBT peptide conjugates, in which BTBT was introduced at the N-terminus separated from the peptide sequence by a flexible linker [32]. These hybrid molecules self-assembled in water to form nanofibers that showed an average conductivity of 4.2 (±1.8) × 10^−6^ S cm^−1^ and 2.4 (±0.47) × 10^−7^ S cm^−1^ for the BTBT-peptide and the C8-BTBT-peptide, respectively. More recently, we reported the first BTBT-peptide hydrogel [33]. We functionalised the N-terminus of a β-sheet self-assembling tetrapeptide containing glutamic acid (Glu) and phenylalanine (Phe), namely Glu-Phe-Glu-Phe, with the BTBT core. Furthermore, hydrogels were obtained by tuning both the pH and the ionic strength of the solution.

As mentioned above, the type of amino acids and their sequence determine the type of self-assembly, and thus, subtle structural variations might lead to changes in the type and strength of the non-covalent interactions involved, also leading to different supramolecular architectures [34,35,36]. For this reason, we aimed to study the effect of introducing the BTBT core in the side chain of the main peptide backbone. To favour a straightforward synthesis of this derivative, the Cu(I)-catalysed azido alkyne cycloaddition (CuAAC) was used to functionalise the amino acid side chain with the BTBT core. An alternating sequence of hydrophilic and hydrophobic amino acids was maintained in order to favour β-sheet formation. Self-supporting transparent hydrogels were obtained upon pH switching. We investigated the solution-based thin film deposition and the electrical properties of this new material.

## 2. Results and Discussion

### 2.1. Design and Synthesis of the BTBT-Peptide Hybrid ***1***

**Scheme 1:**
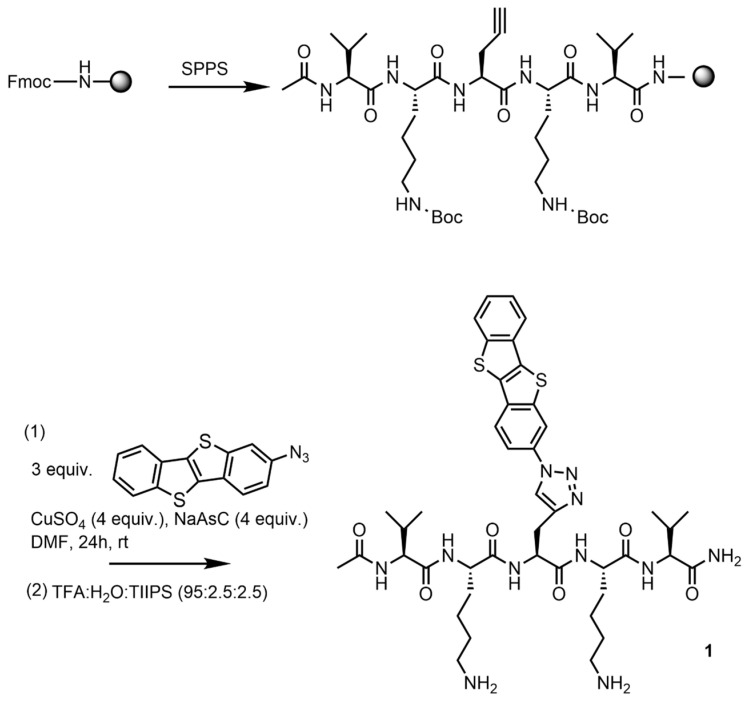
Synthesis of the BTBT-peptide conjugate **1**.

The molecular architecture of the BTBT-peptide hybrid **1** (Scheme 1) alternates hydrophilic and hydrophobic residues in its structure. This alternation favours the formation of β-sheet structures in which the side chains of adjacent residues point out in opposite directions, generating two distinct faces: a hydrophobic and a hydrophilic one. In aqueous media, two sheets may self-assemble to form a β-sheet bilayer in which hydrophobic residues are buried inside, while the hydrophilic faces are exposed to the solvent. It has been demonstrated that this type of self-assembly can accommodate large aromatic side-chains in the inner shielded region and that increasing the area of the aromatic surface leads to stronger π-π interactions [37]. Thus, we expected to obtain self-assembled nanostructures with an aromatic core in which strong π-π interactions are established between BTBT chromophores.

Concerning the peptide design, two valine residues (Val) were positioned at the N- and C-termini, the hydrophilic lysine residues (Lys) enabled pH-dependent solubility in water, while the central position was occupied by the non-natural amino acid L-propargylglycine (Pra) functionalised with BTBT. The N-acetylated peptide sequence was synthesised following fluorenylmethoxycarbonyl (Fmoc) solid-phase peptide synthesis (SPPS) protocols on a 4-methylbenzhydrylamine (MHBA) Rink amide resin. Couplings were carried out using a mixture of O-benzotriazole-N,N,N′,N′-tetramethyluronium hexafluorophosphate (HBTU), 1-Hydroxybennzotriazole hydrate (HOBt), and N,N-diisopropylethylamine (DIPEA). Fmoc deprotection was achieved by treatment with a 20% solution of 4-methylpiperidine in dimethylformamide (DMF). The BTBT moiety was then introduced through an on-resin CuAAC reaction between the terminal alkyne of the Pra side chain and the azide group of 2-azido-BTBT (**2**) in the presence of sodium ascorbate (NaAsc) and CuSO_4_ (Scheme 1). Final cleavage was performed using a trifluoroacetic acid (TFA)/triisopropylsilane (TIPS)/water cocktail (See Supporting Information for details). Compounds **1** and **2** were thoroughly characterised by NMR, FT-IR and ESI-MS (See Figures S1–S8 of the Supporting Information).

### 2.2. Gelation and Hydrogel Characterization

**Figure 1:**
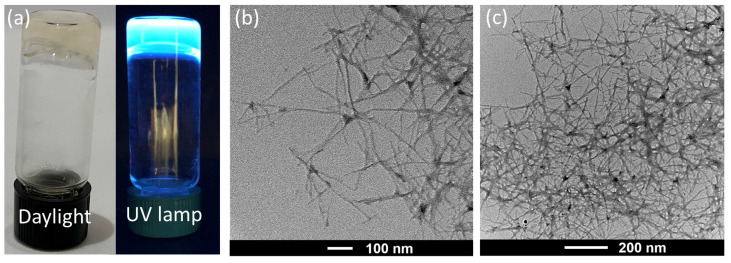
(**a**) Photograph of the gel under daylight and UV lamp; gel formation was assessed by the vial inversion test. (**b**,**c**) TEM micrographs of the xerogels.

**1** is well soluble in acidic water, where the Lys side chains are protonated. When the pH of the solution was raised above 10 by adding NaOH 0.5 N, a self-supporting hydrogel was formed in a few minutes (Figure 1a). The minimum gelation concentration (mgc) was equal to 0.5 wt % and the gel-to-solution transition (Tgel) was observed at 80 °C.

The morphology of the self-assembled nanostructures was analysed by transmission electron microscopy (TEM). TEM micrographs of the xerogel revealed the formation of long-range fibres with an average diameter of 10 (±2) nm and a length of up to 500 nm (Figure 1b,c).

**Figure 2:**
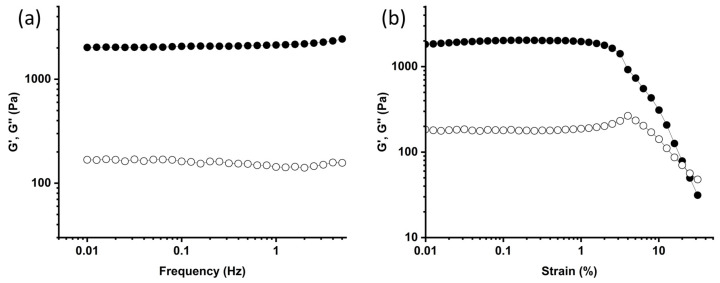
(**a**) Frequency sweep experiment and (**b**) strain sweep experiment of gel at 0.5 wt % concentration; filled circles represent G′ while open circles represent G″.

The gel nature of the sample was confirmed via oscillatory rheology. As expected for viscoelastic materials, frequency sweep experiments showed that the elastic modulus (G′) was one order of magnitude higher than the viscous one (G″) within the linear viscoelastic region, and both contributions were frequency-independent within the investigated range (Figure 2a). In the strain sweep set-up, the viscous and elastic modulus deviated from linearity above 3% of applied strain, reaching the cross-over point at 25% of strain (Figure 2b).

Self-assembly was also investigated by UV-Vis absorption and emission spectroscopies and circular dichroism (CD).

**Figure 3:**
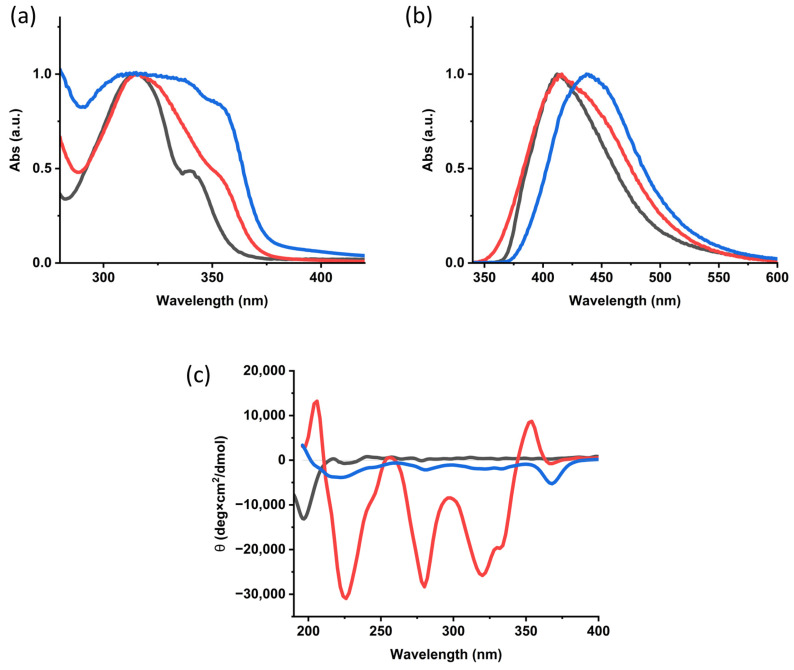
Normalized (**a**) UV-Vis absorption and (**b**) emission spectra and (**c**) CD spectra of **1** in aqueous solution at $10^{-5}$ M (black line), at $5.6\times 10^{-3}$ M (red line) and the gelled sample (blue line).

The UV-Vis absorption spectrum of a 10^−5^ M solution of **1** in MilliQ water showed a structured band going from 285 nm to 350 nm originating from the BTBT core (Figure 3a) [38]. The absorption maximum was centred at 315 nm, with a shoulder located at 340 nm. The emission profile was characterised by an asymmetric band centred at 415 nm with a less-pronounced shoulder at 430 nm. Increasing the concentration to 0.5 wt % (5.6 × 10^−3^ M), a widening of the absorption band of about 12 nm was observed. The maximum absorption peak remained at 315 nm while the less-pronounced shoulder red-shifted to 352 nm. Even the emission spectroscopy showed a broadening of the profile (Figure 3b). The broad emission band showed a maximum at 415 nm, with a shoulder at 440 nm and a long tail up to 620 nm. In an alkaline environment (viz. pH > 10), the gel was formed. The pH-triggered hydrogel showed the broadest UV-Vis absorption spectrum. The main absorption band ranged from 305 nm to 340 nm, while the shoulder increased its intensity and moved to 355 nm. The emission maximum was red-shifted to 445 nm and a tail was observed up to 650 nm.

These data suggest the presence of strong π-π interactions between the BTBT chromophores upon gelation.

This hypothesis was confirmed by CD investigations (Figure 3c). The CD spectrum of a $10^{-5}$ M solution of **1** in water showed a silent CD in the BTBT absorption region and a negative peak at 200 nm that indicates a random coil conformation of the peptide segment. Upon increasing the concentration, new strong negative CD signals emerge at 228 nm and above 280 nm, in the BTBT absorption region, suggesting the formation of β-sheet structures [39] and a chiral arrangement of the chromophores within the aggregate. In the hydrogel, the intensity of the CD signal decreased significantly as a consequence of scattering due to the formation of the 3D fibrillar network. Nevertheless, five negative peaks, located at 368 nm, 332 nm, 320 nm, 280 nm, and 220 nm, could be detected. Signals above 280 nm arise from the BTBT moiety, indicating that the chromophore is involved in the formation of a chiral structure. The negative peak at 220 nm is consistent with the formation of a H-bonded β-sheet network [39].

### 2.3. Electrical Characterization

**Figure 4:**
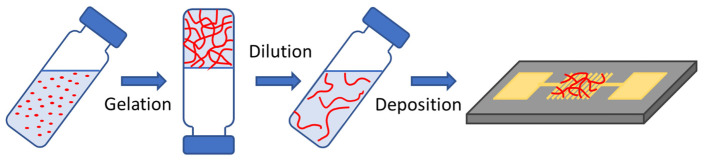
Schematic representation of the deposition process.

In recent years, 2D and 3D conductive hydrogel scaffolds have been extensively studied due to their potential use in tissue engineering applications [40]. We evaluated the charge-transport behaviour of the designed gelator by performing electrical measurements on drop-casted thin films deposited on gold interdigitated electrodes. The hydrogels were first obtained at the mgc concentration and then diluted with water immediately prior to deposition (Figure 4). Optimization of the deposition process showed that the addition of 1% *v*/*v* of N-methyl-2-pyrrolidone (NMP) is necessary to avoid the formation of a “coffee ring” [41]. The addition of NMP did not affect the spatial organisation of the gelator, and the fibrillar structure was maintained, as confirmed by SEM micrographs (Figure S9 of the Supporting Information). Upon drop-casting, the sample was dried at room temperature and then kept under a primary vacuum overnight. Interestingly, we noted that the outcome of the deposition was highly affected by its surrounding. To ensure the reproducibility of the deposition, it was necessary to maintain the NMP-water reservoir close to the BTBT droplets to regulate humidity and allow a control over the drying process. In this context, a few droplets of the 1% NMP solution were placed on the Si/SiO_2_ substrates prior to the deposition of BTBT.

**Figure 5:**
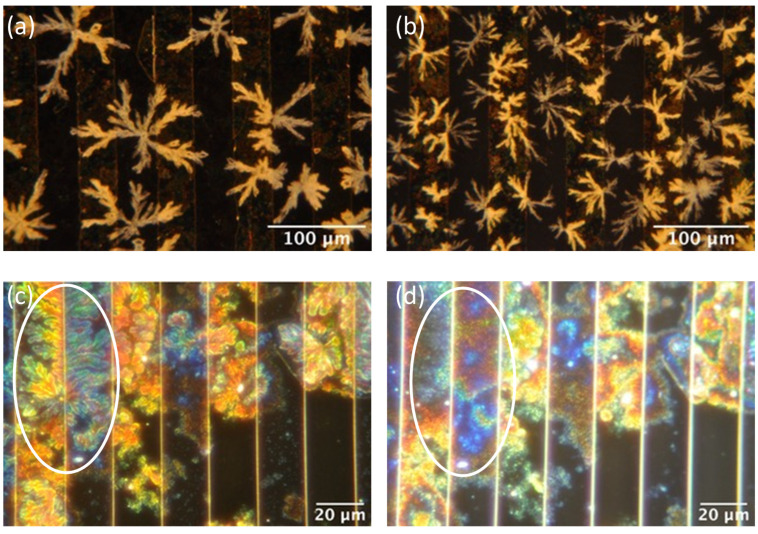
Dark-field optical microscope images of **1** deposited on gold interdigitated electrodes (**a**,**b**). (**c**,**d**) Dark- field optical microscope images before and after rising with water, respectively. White circle highlights the removal of adsorbed dendrimeric shapes.

This specific deposition allowed the control and formation on top of the electrodes of dendrimeric features, as shown in Figure 5a–c. These particular structures were able to electrically bridge the interdigitated electrodes. These fibrillar structures are physically adsorbed, hence they can be completely removed by rinsing the substrate with water (Figure 5c,d).

**Figure 6:**
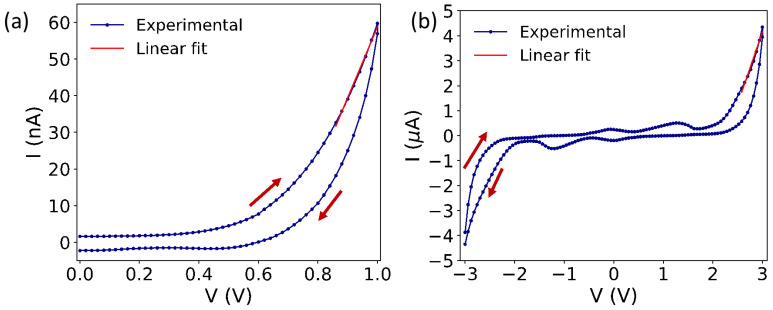
I-V plots of the BTBT onto Au IDEs in which the applied voltage swept from (**a**) 0 V to +1 V, and (**b**) −3 V to +3 V.

Electrical characterisation was performed by recording the I-V characteristics of the BTBT devices in the air. Figure 6a,b present the I-V profile obtained when the voltage was swept from 0 V to +1 V, and from −3 V to +3 V, respectively. In Figure 6a, it is possible to observe a rectifying behaviour due to the p-n diodes composed by the Au electrode and the BTBT film [32]. This trend is ruled by Equation (1):

(1) $$I=I_{0}\left[\exp\left(\frac{V}{A}\right)-1\right],$$

in which I_0_ corresponds to the reverse bias current, and A is a linear function of the ideality factor and the thermal voltage. Within this context, the first derivative of current with respect to the applied voltage indicates a diode-limited current that evolves to a resistor-limited current when the applied voltage approaches 1 V. In the resistive region, the linear fit provides a conductivity of 5.3 (±0.1) × 10^−7^ S cm^−1^, which has the same order of magnitude as that presented in the literature for C8-BTBT-peptide films. Nevertheless, as our device works in a voltage range that is twenty times lower, it opens up the possibility of being applied on low-power devices. We also investigated the electrical response of the BTBT devices in a broader voltage range, as shown in Figure 6b. In this case, a peak was observed at +1.3 V and −1.2 V. Such a feature has already been reported in the literature for the cyclic voltammetry of BTBT in 1 mM dichloromethane, and it was attributed to reversible redox of the BTBT compounds [42]. However, as our measurements are performed in the air, we attribute the presence of such redox activity to the presence of trapped water in the BTBT compound, which allows for charge transport. The superposition of these peaks to the diode profile causes a reduction in the measured current in the resistive region according to consecutive scans. The stability of these devices was achieved by leaving the samples in vacuum overnight and by shielding them with a drop-casted PMMA layer. The PMMA layer also prevented the detachment of dendrimeric features from the substrate. Figure S10 of the Supporting Information illustrates the improved performance for six consecutive scans. Under these conditions, the resistive region provides a conductivity of 1.6 (±0.1) × 10^−5^ S cm^−1^. This value is one order of magnitude higher than the conductivity reported previously for other non-gelled assemblies of BTBT-peptide hybrids [32].

## 3. Materials and Methods

Preparative high performance liquid chromatography (HPLC): This was performed using an ÄKTA Pure GE Healthcare apparatus equipped with a Jupiter C18 (250 × 22 mm, 10 μm, 300 Å) column. The UV SPD-20A detector was used at 217 nm, flow rate 15 mL/min, in a binary elution system (solvent A: H_2_O + 0.05% TFA; solvent B: acetonitrile + 0.05% TFA).

Analytical HPLC-MS: HPLC-MS analysis was performed by using an HPLC Agilent Technologies 1260 Infinity II equipped with a KINETEX XB C18 (100 × 4.60 mm, 100 Å, 3.5 μm) column. HPLC was connected with an Agilent Technologies 6130 Quadrupole LC/MS instrument. Solvent A: H_2_O + 0.05% TFA; solvent B: ACN + 0.05% TFA.

Nuclear Magnetic Resonance (NMR): ^1^H and ^13^C NMR spectra were collected at room temperature on Bruker Avance II 300 or Bruker 400 spectrometers. Chemical shifts (δ) were reported in parts per million (ppm). The signal of the partially deuterated solvent was used as internal standard. The signal multiplicity was indicated as s (singlet), d (doublet), t (triplet), m (multiplet), and br (broad).

Fourier Transform Infrared Spectroscopy (FT-IR): FT-IR spectra were recorded using a Perkin-Elmer 720× spectrophotometer at a nominal resolution of 2 cm^−1^ with an average scan of 100.

UV-Vis absorption spectroscopy: UV-Vis absorption spectra were registered using a Varian Cary 50 spectrophotometer at room temperature in a baseline correction mode. Rectangular cells with an optical path of 10 mm or 1 mm were used for the diluted and concentrated solution, respectively. For gelled samples, a detachable window cell with 0.2 mm optical path was employed. All gels were previously prepared in a vial and then transferred to the quartz chamber, which was carefully closed to avoid the formation of bubbles.

Emission spectroscopy: Emission spectra were registered using a Varian CaryEclipse spectrophotometer at room temperature. Rectangular cells with an optical path of 10 × 10 mm were used for diluted solutions, while concentrated solutions and gels were analysed using a cell with a 10 × 4 mm optical path. For gelled samples, the gel was directly prepared within the quartz cell and analysed without amendment.

Circular Dichroism (CD) Spectroscopy: CD spectra were collected using a Jasco J-1500 spectropolarimeter at room temperature, as an average of 32 measurements. Baselines were recorded for all spectra. Spectra were reported in terms of total molar ellipticity (deg × cm^2^ × dmol^−1^). The diluted solutions were analysed using a cuvette cell of 10 mm path length, while concentrated solutions were analysed in a 1 mm path length cuvette. Gelled samples were previously prepared in a glass vial and then transferred to a cell with detachable windows and an optical path of 0.2 mm without amendment, and avoiding bubble formation.

Transmission Electron Microscopy (TEM): TEM analysis was performed using a Jeol 300PX TEM instrument. Glow-discharged carbon-coated grids were used. The grid was floated on a small drop of the sample and the excess was removed by Whatman filter paper, grade 50, hardened. Gels were diluted prior to the analysis. The micrographs were analysed using the ImageJ program.

Rheology: Rheological investigations were performed on a Kinexus Lab+ rheometer at room temperature with an anti-evaporation chamber to prevent sample dryness. Gels were prepared in a mould with 2 cm diameter and transferred onto the plate immediately before the analysis. Two-parallel-plated geometry was used for the analysis. Frequency sweep experiments were carried out between 5 Hz and 0.001 Hz at a constant 0.2% strain. Strain sweep experiments were carried out at a constant frequency of 0.2 Hz, varying the strain between 0.01% and 100%.

Electrical measurements: Gold interdigitated electrodes (IDEs), deposited on Si/SiO_2_ and composed of 11 digits having 733 µm length, 40 μm width, and spaced 40 μm from each other (cell constant ≈ 3665 µm), were produced by standard photolithography and used for the electrical characterizations. Samples of 1 mg/mL jellified BTBT were dispersed in bi-distilled water at a concentration of 0.2 mL/mL. The obtained dispersion was then drop-casted onto the IDEs in a 1% NMP atmosphere. The dried samples were put in a vacuum overnight and covered by the drop-casting deposition of 5% PMMA in anisole (*w*/*v*). Electrical characterizations were performed in a Faraday cage in the air and in the dark. An Agilent B1500 parameter analyser equipped with two high-power and two high-resolution source measurement units (SMUs) was used for the I-V characterization.

Synthesis of compound **1**: **1** was achieved using standard SPPS protocols followed by CuCAAC reaction and peptide cleavage (Details can be found in the Supporting Information). On-resin CuAAC reaction: The resin was transferred into a vial and a solution of **2** (3 equiv.) in degassed DMF (10 mL per 1 g of resin) was added, followed by CuSO_4_ (4 equiv.), L-sodium ascorbate (NaAsc, 4 equiv.) and DIPEA (9 equiv). The mixture was shaken for 24 h at room temperature and the reaction was monitored via HPLC. When the reaction was completed, the mixture was transferred to an SPPS reaction vessel and the resin was washed with DMF (×6), H_2_O (×6), Ethylenediaminetetraacetic acid (EDTA, 0.1 M, pH = 6, ×5), H_2_O (×6), and DMF (×6). ^1^H-NMR (400 MHz, DMSO-d6) δ (ppm), 8.748 (d, *J* = 2.0 Hz, 1H), 8.625 (s, 1H), 8.291 (d, *J* = 8.6 Hz, 1H), 8.240 (d, *J* = 7.3 Hz, 1H), 8.205 (d, *J* = 7.2 Hz, 1H), 8.147–8.094 (m, 3H), 8.074 (dd, *J* = 8.6, 2.0 Hz, 1H), 7.831 (d, *J* = 8.2 Hz, 1H), 7.804–7.706 (m, 7H), 7.609–7.490 (m, 2H), 7.415 (s, 1H), 7.081 (s, 1H), 4.634 (q, *J* = 7.3 Hz, 1H), 4.294 (d, *J* = 6.7 Hz, 1H), 4.254–4.023 (m, 3H), 3.234 (dd, *J* = 15.1, 5.2 Hz, 1H), 3.070 (dd, *J* = 15.1, 8.3 Hz, 1H), 2.813–2.690 (m, 4H), 2.035–1.798 (m, 1H), 1.865 (s, 3H), 1.773–1.457 (m, 8H), 1.400–1.240 (m, 4H), 0.887–0.730 (m, 12H). ^13^C NMR (100 MHz, DMSO-d6) δ (ppm), 172.80, 171.54, 171.48, 171.31, 170.41, 169.59, 144.23, 142.65, 141.85, 134.26, 134.08, 132.36, 132.19, 132.06, 125.94, 125.59, 124.64, 122.85, 121.92, 121.36, 117.85, 115.79, 58.10, 57.51, 52.74, 52.62, 52.05, 38.64, 31.16, 30.83, 30.45, 30.14, 26.61, 26.52, 22.49, 22.27, 22.20, 19.24, 19.19, 18.21, 17.91. ESI-MS: [M+H]^+^ calculated for C_43_H_59_N_11_O_6_S_2_ 890.14, found 890.5. [M+Na]^+^ calculated for C_43_H_59_N_11_O_6_S_2_ 912.14, found 912.5. FT-IR (KBr): ν(cm^−1^) = 3407, 3285, 3074, 2963, 2932, 2664, 1674, 1628, 1527, 1430, 1340, 1300, 1243, 1200, 1180, 1132, 1041, 992, 950, 838, 798, 748, 721, 703, 600.

Synthesis of **2**: 2-amino-[1]benzothieno[3,2-b][1]benzothiophene [43] (1.2 g, 4.73 mmol), previously ground, was dispersed in HCl 6 M (27 mL) and heated at 70 °C for 20 min. The reaction mixture was cooled to room temperature and then to 0 °C by using an ice/water bath. A cooled aqueous solution of NaNO_2_ (420 mg, 6.08 mmol in 12.6 mL MilliQ water) was added dropwise. The suspension was stirred for 30 min at 0 °C. Next, the reaction mixture was added dropwise to a solution of NaN_3_ (426 mg, 6.55 mmol in 12.6 mL MIlliQ water) cooled to 0 °C. The mixture was stirred at the same temperature for 2 h, then the aqueous phase was extracted with dichloromethane (DCM) (3×), washed with water and brine, dried over NaSO_4_, and filtered and evaporated to give a brownish solid that was used in the next step without further purification (yield 76%). ^1^H NMR (300 MHz, DMSO-d_6_) δ (ppm), 8.22–7.93 (m, 4H), 7.49 (m 2H), 7.26 (dd, *J* = 8.5, 2.1 Hz, 1H). ^13^C NMR (75 MHz, DMSO-d_6_) δ (ppm), 143.24, 141.50, 137.08, 132.72, 132.48, 132.31, 129.68, 125.48, 125.41, 124.47, 122.87, 121.59, 117.38, 114.69. FT-IR: ν(cm^−1^) 3431, 2107, 1552, 1465, 1339, 1284, 951, 809, 748.

Gel formation: 5 mg of **1** were dissolved in MilliQ water (900 μL) with the aid of sonication. The addition of NaOH (0.5 M, 3 equiv.) and MilliQ water allowed the obtaining of a self-supporting hydrogel with the desired final concentration of 5 mg/mL. Gel formation was assessed via a vial inversion test.

## 4. Conclusions

In summary, we successfully designed and synthesised a hybrid peptide, whose backbone incorporated the [1]benzothieno[3,2-b]benzothiophene (BTBT) small-molecule semiconductor. The hybrid peptide was effectively self-assembled in water to give self-supporting hydrogels upon pH-switching with a fibrillar structure. Spectroscopic studies confirmed the formation of β-sheet assemblies and revealed the presence of strong π-π interactions between BTBT cores, that result in π-delocalization. Such hybrid peptides showed high conductivity values, as high as 5.3 (±0.1) × 10^−7^ S cm^−1^, corresponding to low operational voltages (i.e., 0 < V < 1 V). Broadening the voltage range (−3 V < V < +3 V), we reached a conductivity value equal to 1.6 (±0.1) × 10^−5^ S cm^−1^. To the best of our knowledge, this conductivity value is one order of magnitude higher than state-of-the-art values reported in the literature related to non-gel BTBT-peptide hybrids. Such promising results pave the way towards interesting applications, where a soft, nanostructured, electrically-active and biocompatible material is required.

## Data Availability

The data presented in this study are available in the article and/or supplementary material.

## Supplementary Materials

The following supporting information can be downloaded at: https://www.mdpi.com/article/10.3390/molecules28072917/s1, Detailed synthetic procedures and characterization of **1** and **2** [43]. Figure S1: ^1^H NMR (DMSO-d6, 300 MHz) of **2**, Figure S2: ^13^C NMR (DMSO-d6, 75 MHz) of **2**, Figure S3: ^1^H NMR (DMSO-d6, 400 MHz) of **1**, Figure S4: ^13^C-NMR (DMSO-d6, 100 MHz) of **1**, Figure S5: FT-IR spectrum of **2** (KBr disk), Figure S6: FT-IR spectrum of **2** (KBr disk), Figure S7: HPLC chromatogram of **1**, Figure S8: ESI-MS of **1**, Figure S9: SEM micrographs of the gel, Figure S10: Consecutive I-V measurements of the BTBT-peptide onto Au IDEs in which the applied voltage swept from −3 V to +3 V.
