# Supplementary material for: Self-Assembly and Electrical Conductivity of a New [1]benzothieno[3,2-b][1]-benzothiophene (BTBT)-Peptide Hydrogel
Source: Molecules. 2023 Mar 24;28(7):2917. doi: 10.3390/molecules28072917 (PMC10095725; doi:10.3390/molecules28072917)
Supplement: Supplementary file 1 [file molecules-28-02917-s001.zip › molecules-2281770-supplementary-revised.pdf]

Supporting Information

Self-assembly and electrical conductivity of a new [1]benzothieno[3,2-b][1]-benzothiophene (BTBT)-peptide hydrogel

Anna Fortunato, Rafael Cintra Hensel, Stefano Casalini and Miriam Mba*

SYNTHETIC PROCEDURES

Rink Amide MBHA Resin was purchased from Irish Biotech (commercial loading 0.68 mmol/g). Fmoc-amino acids were purchased from Merck or Iris Biotech. All other reagents and solvents were purchased from Merck and used without further purifications. 2-amino-[1]benzothieno[3,2-b][1]benzothiophene was synthesized as previously reported [1].

Preparative high performance liquid chromatography (HPLC): was performed using ÄKTA Pure GE Healthcare apparatus equipped with a Jupiter C18 (250 x 22 mm, 10μm, 300Å) column. The UV SPD-20A detector was used at 217 nm, flow rate 15 mL/min in a binary elution system (solvent A: H_2O+ 0.05% trifluoroacetic acid (TFA); solvent B: acetonitrile (ACN)+0.05% TFA).

Analytical HPLC-MS: HPLC-MS analysis was performed by using HPLC Agilent Technologies 1260 Infinity II equipped with KINETEX XB C18 (100 x 4,60 mm, 100Å, 3,5 μm) column. HPLC was connected with Agilent Technologies 6130 Quadrupole LC/MS instrument. Solvent A: H_2O+ 0.05% TFA; solvent B: ACN+0.05% TFA.

Nuclear Magnetic Resonance (NMR): ^{1}H and ^{13}C NMR spectra were collected at room temperature on Bruker Avance II 300 or Bruker 400 spectrometers. Chemical shifts (δ) are reported in parts per million (ppm). The signal multiplicity is indicated as s (singlet), d (doublet), t (triplet), m (multiplet) and br (broad).

Fourier Transform Infrared Spectroscopy (FT-IR): FT-IR spectra were recorded using a Perkin-Elmer 720X spectrophotometer at a nominal resolution of 2 cm^{-1} with an average scan of 100.

[1] Košata, B.; Kozmík, V.; Svoboda, J. Reactivity of [1]Benzothieno[3,2-b][1]benzothiophene - Electrophilic and Metallation Reactions. *Collection of Czechoslovak Chemical Communications* **2002**, *67*, 645-664.

Synthesis of 2-azido-[1]benzothieno[3,2-b][1]benzothiophene (2)

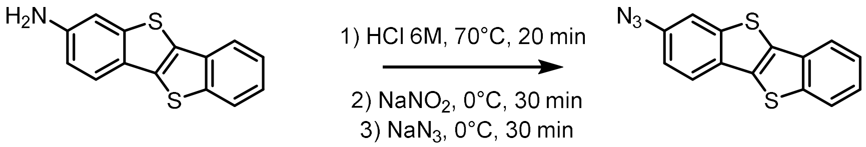

2-amino-[1]benzothieno[3,2-b][1]benzothiophene (1.2 g, 4.73 mmol), previously ground, was dispersed in HCl 6M (27 mL) and heated at 70°C for 20 minutes. The reaction mixture was cooled to room temperature and then to 0°C by using an ice/water bath. A cooled aqueous solution of $NaNO_2$ (420 mg, 6.08 mmol in 12.6 mL MilliQ water) was added dropwise. The suspension was stirred for 30 minutes at 0°C. Next, the reaction mixture was added dropwise to a solution of NaN_3 (426 mg, 6.55 mmol in 12.6 mL MIlliQ water) cooled at 0°C. The mixture was stirred at the same temperature for 2 hours, then the aqueous phase was extracted with dichloromethane (DCM) (3x), washed with water and brine, dried over $NaSO_4$, filtered and evaporated to give a brownish solid that was used in the next step without further purification (yield 76%).

^{1}H NMR (300 MHz, DMSO-d_6) δ (ppm) 8.22-7.93 (m, 4H), 7.49 (m 2H), 7.26 (dd, *J* = 8.5, 2.1 Hz, 1H).

^{13}C NMR (75 MHz, DMSO-d_6) δ (ppm) 143.24, 141.50, 137.08, 132.72, 132.48, 132.31, 129.68, 125.48, 125.41, 124.47, 122.87, 121.59, 117.38, 114.69.

FT-IR: ν(cm^{-1}) 3431, 2107, 1552, 1465, 1339, 1284, 951, 809, 748

Solid phase peptide synthesis of 1.

When not used the resin was dried and stored in fridge with the N-terminus Fmoc-protected. The synthesis was performed in a manual glass vessel equipped with a glass frit and two outlets. All the synthetic steps were carried out under N2 that was bubbled from below to get stirring. A washing step implies 1 min of stirring and then removal of solvent. In general, 10 mL of solvent must be used for 1 gram of resin.

- The resin was swelled with dimethylformamide (DMF) (10 mL per gram of resin) for 40 minutes prior to use.
- Fmoc deprotection was performed by treating the resin with a solution of 20% of piperidine in DMF for 15 minutes. The step was repeated twice and the resin was washed with DMF (x3).
- Amino acid coupling: the amino acids were externally activated by mixing Fmoc-protected amino acid (3 equiv.), O-Benzotriazole-N,N,N',N'-tetramethyluronium hexafluorophosphate (HBTU) (2.9 equiv.), 1-Hydroxybenzotriazole hydrate (HOBt) (2.9 equiv.) and N,N-Diisopropylethylamine (DIPEA) (9 equiv.) in DMF, then added to the peptide synthesis vessel containing the resin and stirred for 1 hour. The resin was washed with DMF (x3). All coupling steps were monitored using a Kaiser test). If necessary, the coupling step was repeated.
- Acetylation of the N-terminal: upon completion of the amino acid sequence, the Fmoc group of the N-terminus was deprotected as described above and then treated with a mixture of Ac_2O/DIPEA/DMF 1:2:7 for 40 minutes. The resin was washed with DMF (x3). The reaction was monitored using a Kaiser test on a few resin beads which were removed from the peptide chamber after drying with DCM. If necessary, the acetylation step was repeated.
- On resin CuAAC reaction: The resin was transferred into a vial and a solution of **2** (3 equiv.) in degassed DMF (10 mL *per* 1 g of resin) was added, followed by $CuSO_4$ (4 equiv.), L-Sodium ascorbate (NaAsc, 4 equiv.) and DIPEA (9 equiv). The mixture was shaken for 24 hours at room temperature and the reaction was monitored *via* HPLC. When the reaction was completed, the mixture was transferred to a SPPS reaction vessel and the resin was washed with DMF (x6), H_2O (x6), Ethylenediaminetetraacetic acid (EDTA, 0.1M, pH=6, x5), H_2O (x6) and DMF (x6).
- Peptide cleavage: Cleavage was performed in a SPPS reaction vessel. The resin was treated with a mixture of trifluoroacetic acid, water and triisopropylsilane (TFA:H_2O:TIPS 95:2.5:2.5) for 3 hours. The solvents were collected in a flask. The resin was washed with DCM (4x) and this solvent was also collected. Collected solvents were evaporated *via* rotavapor to dryness and the product was precipitated from cold diethyl ether and collected by centrifugation. Subsequently, the peptide was dissolved in water and lyophilized before purification by preparative HPLC. The purified fraction was lyophilized from HCl solution (0.05M) thrice to enable the counter-ion exchange.

^{1}H-NMR (400 MHz, DMSO-d6) δ (ppm) 8.748 (d, *J* = 2.0 Hz, 1H), 8.625 (s, 1H), 8.291 (d, *J* = 8.6 Hz, 1H), 8.240 (d, *J* = 7.3 Hz, 1H), 8.205 (d, *J* = 7.2 Hz, 1H), 8.147 – 8.094 (m, 3H), 8.074 (dd, *J* = 8.6, 2.0 Hz, 1H), 7.831 (d, *J* = 8.2 Hz, 1H), 7.804 – 7.706 (m, 7H), 7.609 – 7.490 (m, 2H), 7.415 (s, 1H), 7.081 (s, 1H), 4.634 (q, *J* = 7.3 Hz, 1H), 4.294 (d, *J* = 6.7 Hz, 1H), 4.254 – 4.023 (m, 3H), 3.234 (dd, *J* = 15.1, 5.2 Hz, 1H), 3.070 (dd, *J* = 15.1, 8.3 Hz, 1H), 2.813 – 2.690 (m, 4H), 2.035 – 1.798 (m, 1H), 1.865 (s, 3H), 1.773 – 1.457 (m, 8H), 1.400 – 1.240 (m, 4H), 0.887 – 0.730 (m, 12H).

^{13}C NMR (100 MHz, DMSO-d6) δ (ppm) 172.80, 171.54, 171.48, 171.31, 170.41, 169.59, 144.23, 142.65, 141.85, 134.26, 134.08, 132.36, 132.19, 132.06, 125.94, 125.59, 124.64, 122.85, 121.92, 121.36, 117.85, 115.79, 58.10, 57.51, 52.74, 52.62, 52.05, 38.64, 31.16, 30.83, 30.45, 30.14, 26.61, 26.52, 22.49, 22.27, 22.20, 19.24, 19.19, 18.21, 17.91.

ESI-MS: $[M+H]^+$ calculated for $C_{43}H_{59}N_{11}O_6S_2$ 890.14, found 890.5. $[M+Na]^+$ calculated for $C_{43}H_{59}N_{11}O_6S_2$ 912.14, found 912.5.

FT-IR (KBr): ν(cm^{-1}) = 3407, 3285, 3074, 2963, 2932, 2664, 1674, 1628, 1527, 1430, 1340, 1300, 1243, 1200, 1180, 1132, 1041, 992, 950, 838, 798, 748, 721, 703, 600.

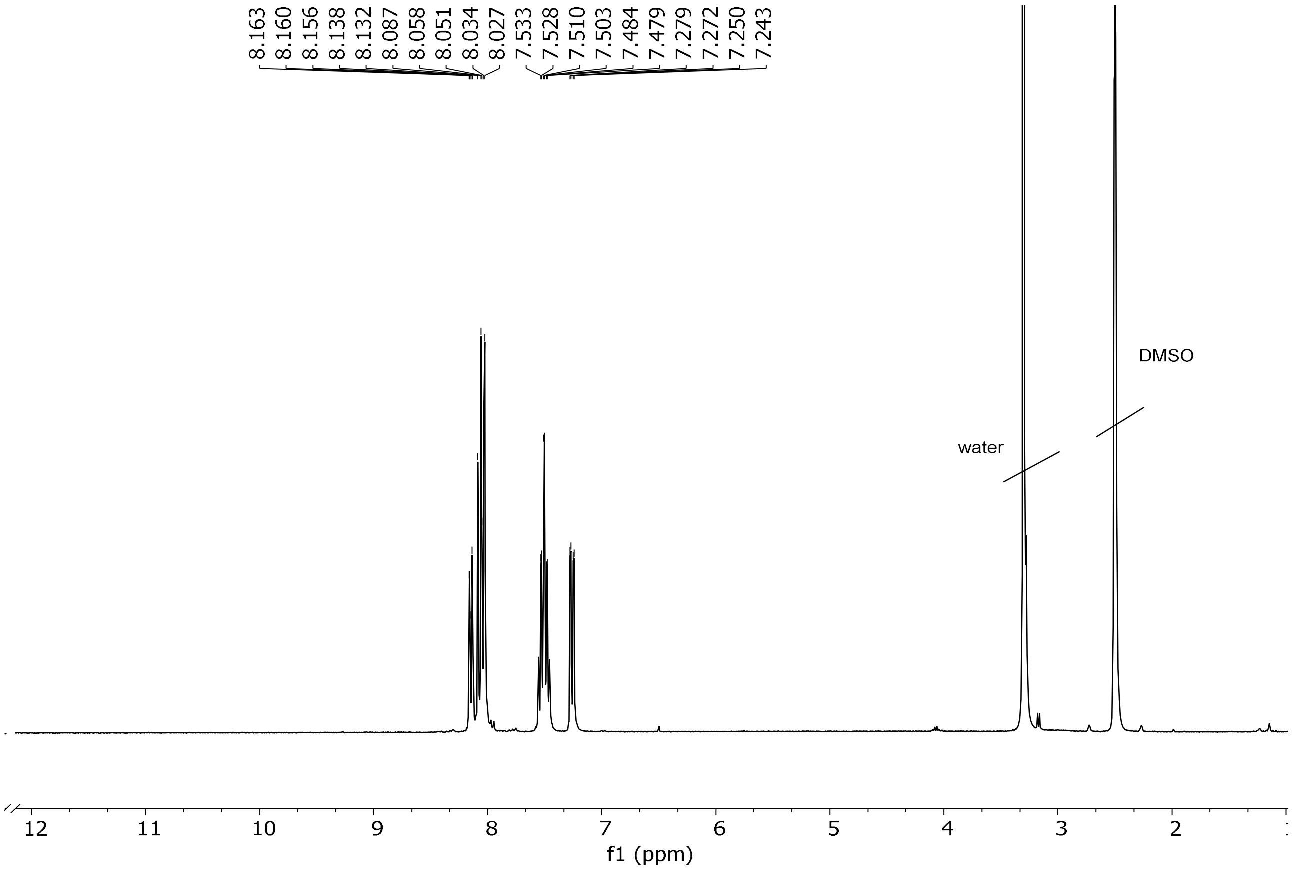

Figure S1. ^{1}H NMR (300 MHz, DMSO-d_6) of **2**.

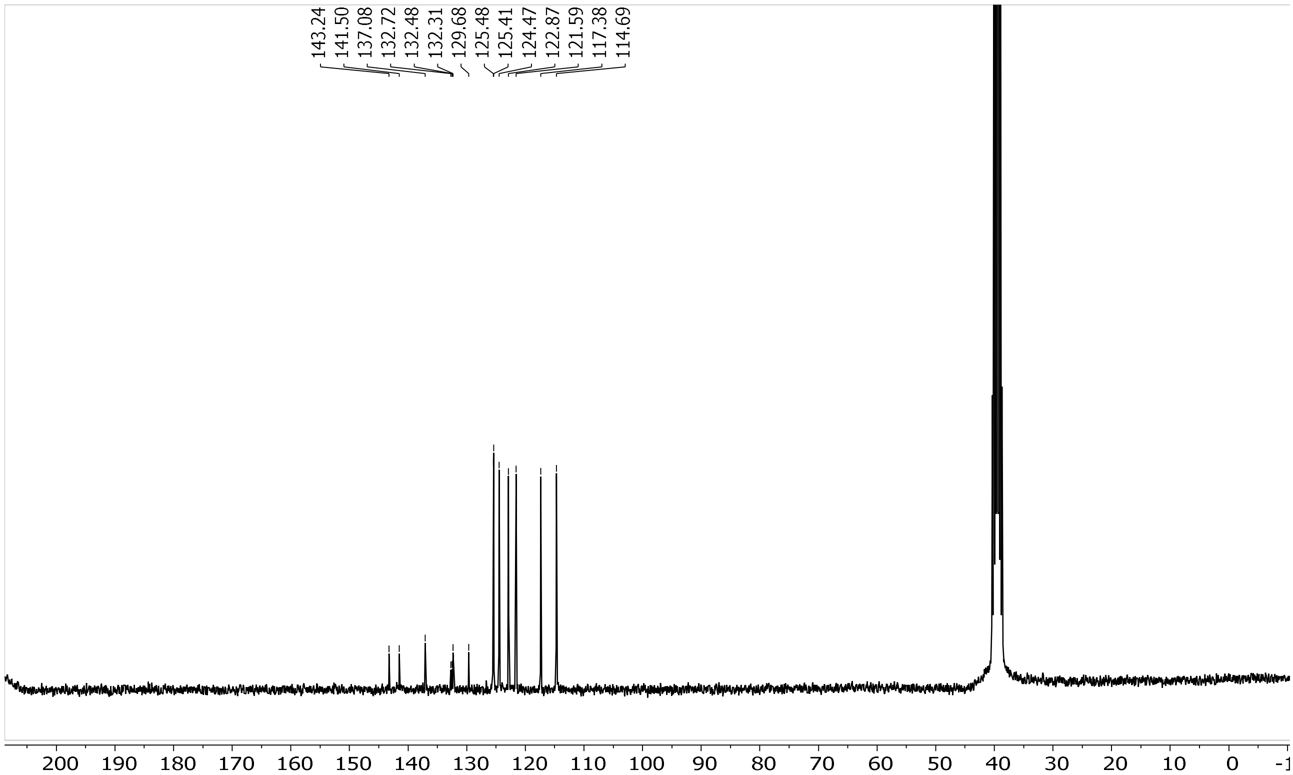

Figure S2. ^{13}C NMR (75 MHz, DMSO-d_6) of **2**.

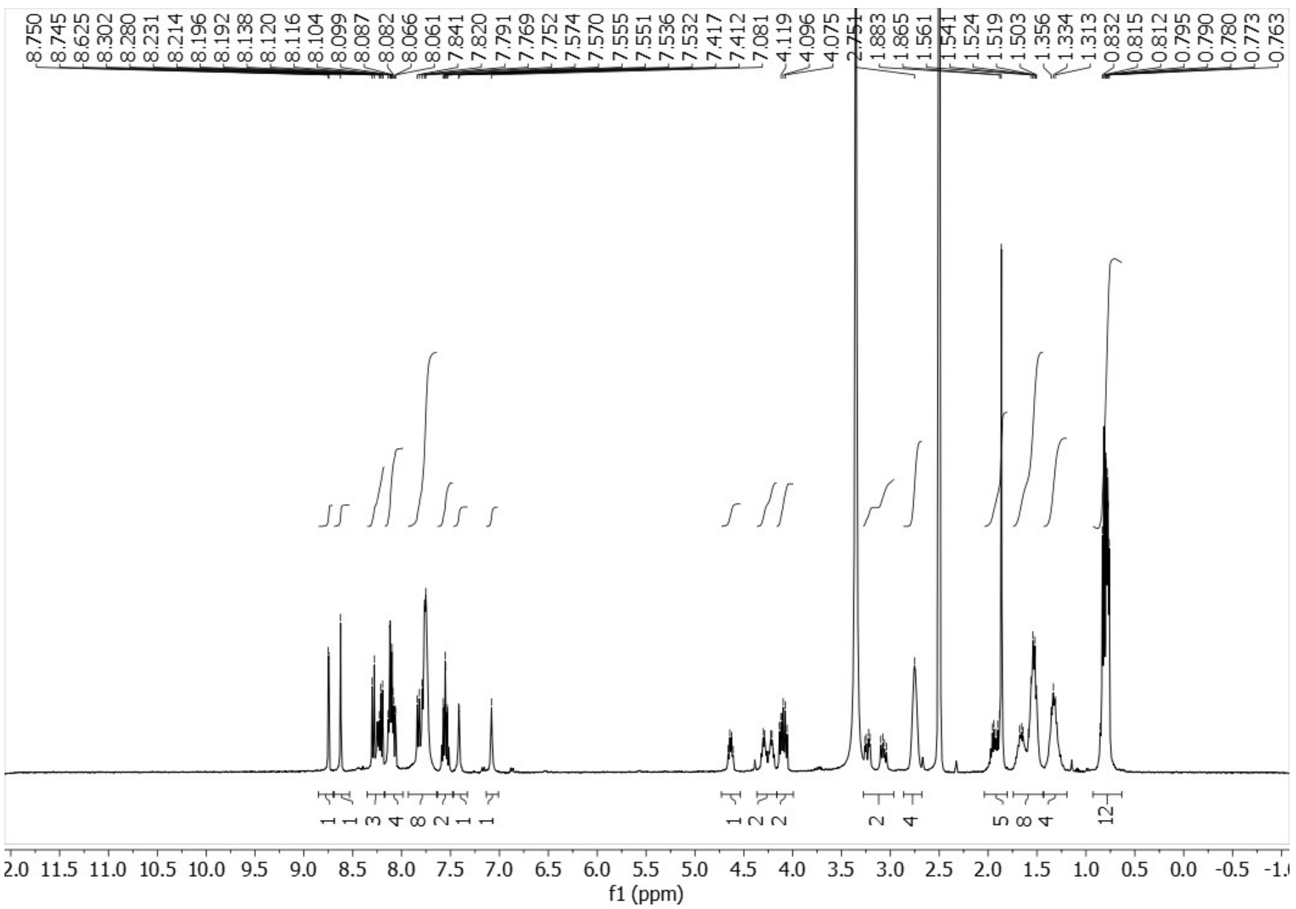

Figure S3: ^{1}H NMR (400 MHz, DMSO-d$_6$) of **1**.

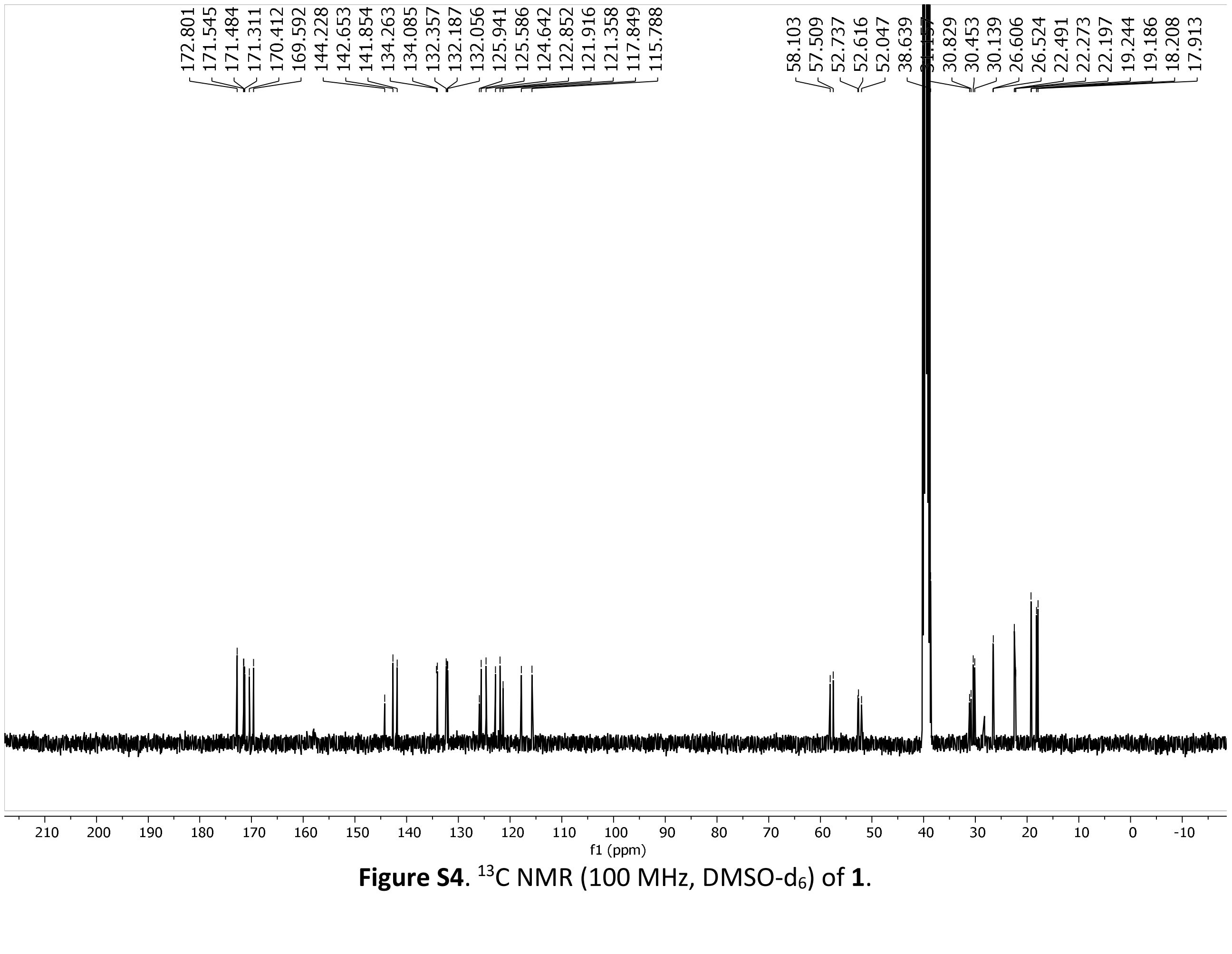

Figure S4. ^{13}C NMR (100 MHz, DMSO-d$_6$) of **1**.

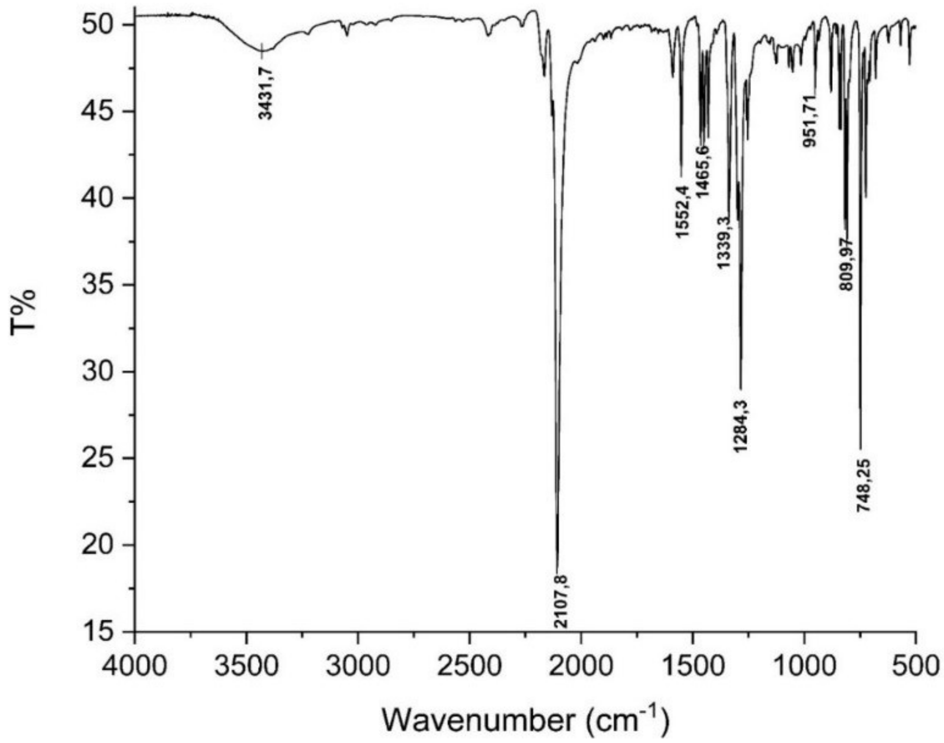

Figure S5. FT-IR (KBr disc) of **2**.

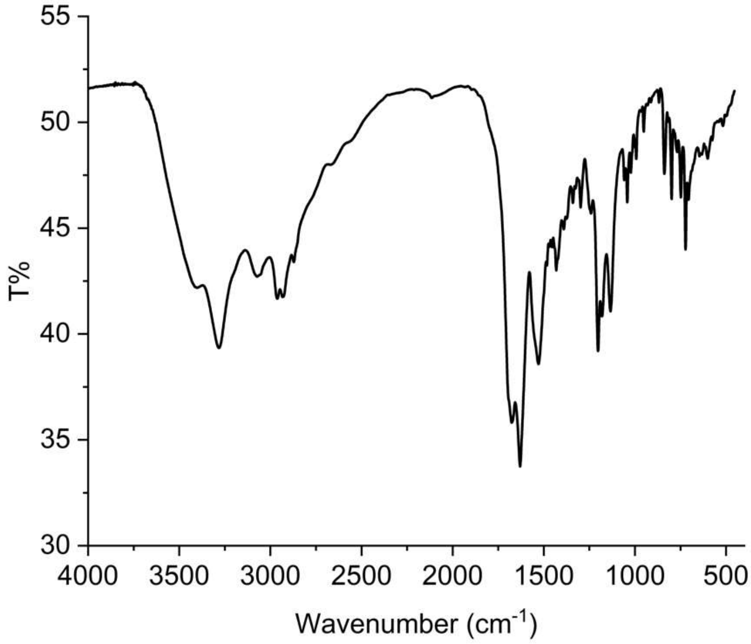

Figure S6: FT-IR spectrum of **1** powder in KBr disc.

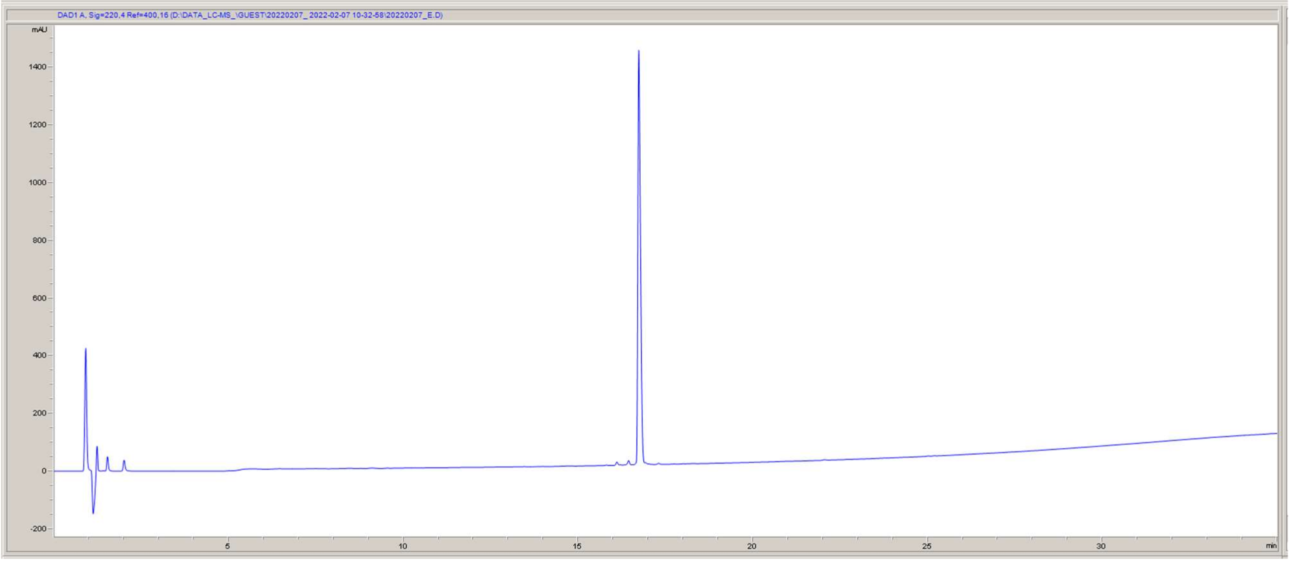

Figure S7. HPLC chromatograph of **1** upon purification, purity 99%.

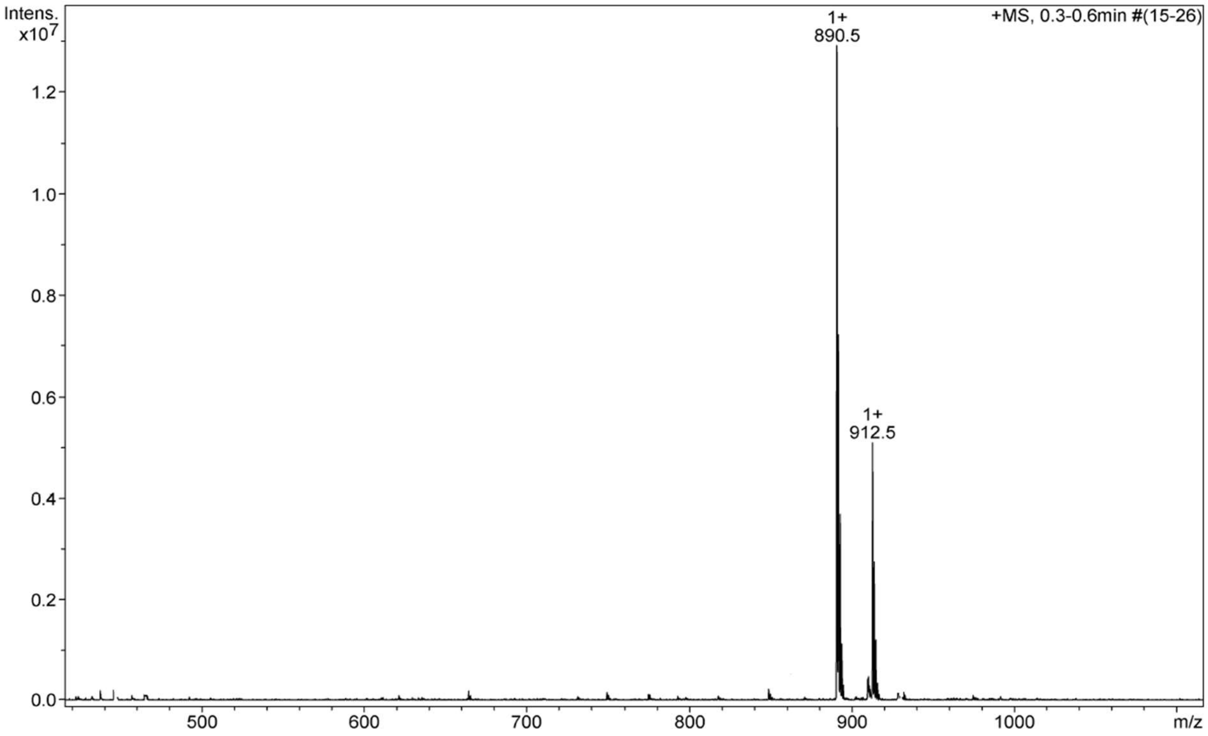

Figure S8. ESI-MS profile of **1** after purification.

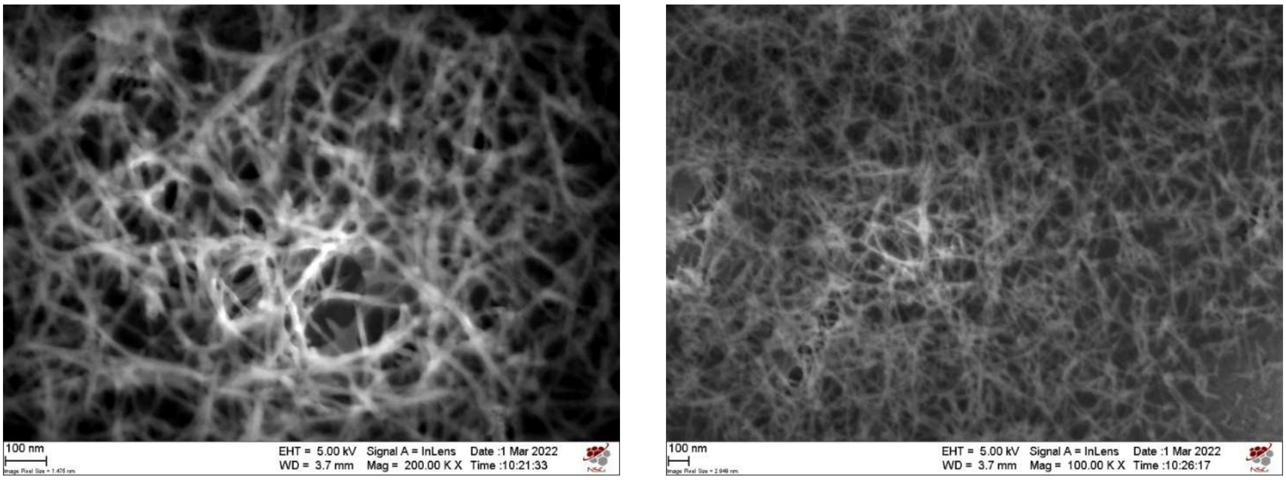

Figure S9. SEM micrographs of the gel.

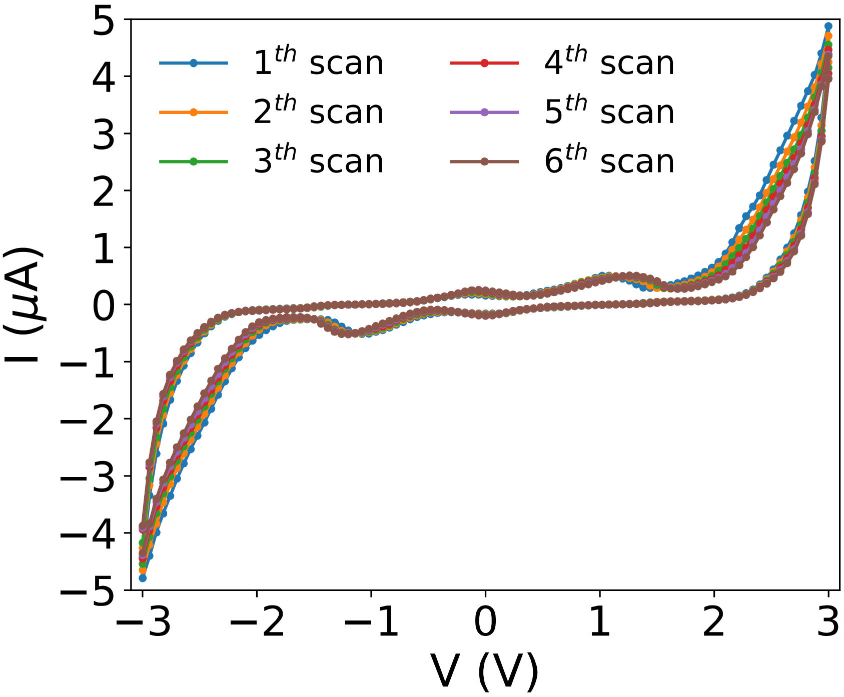

Figure S10. Consecutive I-V measurements of the BTBT-peptide onto Au IDEs in which the applied voltage swept from -3 V to +3V.
